# Personalized chemotherapy selection for breast cancer using gene expression profiles

**DOI:** 10.1038/srep43294

**Published:** 2017-03-03

**Authors:** Kaixian Yu, Qing-Xiang Amy Sang, Pei-Yau Lung, Winston Tan, Ty Lively, Cedric Sheffield, Mayassa J. Bou-Dargham, Jun S. Liu, Jinfeng Zhang

**Affiliations:** 1 Department of Statistics, Florida State University, Tallahassee, FL, 32306, USA; 2 Department of Chemistry & Biochemistry, Florida State University, Tallahassee, FL, 32306, USA; 3 Mayo clinic, 4500 San Pablo Rd S, Jacksonville, FL, 32224, USA; 4 Department of Statistics, Harvard University, Cambridge, MA, 02138, USA

## Abstract

Choosing the optimal chemotherapy regimen is still an unmet medical need for breast cancer patients. In this study, we reanalyzed data from seven independent data sets with totally 1079 breast cancer patients. The patients were treated with three different types of commonly used neoadjuvant chemotherapies: anthracycline alone, anthracycline plus paclitaxel, and anthracycline plus docetaxel. We developed random forest models with variable selection using both genetic and clinical variables to predict the response of a patient using pCR (pathological complete response) as the measure of response. The models were then used to reassign an optimal regimen to each patient to maximize the chance of pCR. An independent validation was performed where each independent study was left out during model building and later used for validation. The expected pCR rates of our method are significantly higher than the rates of the best treatments for all the seven independent studies. A validation study on 21 breast cancer cell lines showed that our prediction agrees with their drug-sensitivity profiles. In conclusion, the new strategy, called PRES (Personalized REgimen Selection), may significantly increase response rates for breast cancer patients, especially those with HER2 and ER negative tumors, who will receive one of the widely-accepted chemotherapy regimens.

Breast cancer is a heterogeneous disease. Previously, validated clinicopathologic prognostic factors, such as tumor size, lymph node involvement, and histologic grade, have been widely used by clinicians to guide treatment decisions. This approach resulted in significant numbers of overtreated and undertreated patients. More recently, evaluation of the status of estrogen receptor (ER), human epidermal growth factor receptor 2 (HER2), and progesterone receptor (PR) has become routine practice because they have been validated as prognostic markers and drug targets. The development of high-throughput genomics technologies (*e.g*., microarrays and next generation sequencing) has enabled even more specific personalized cancer therapy (PCT)^1^,^2^,^3^. When using patients’ genomic profiles, a set of markers needs to be selected and often combined with clinical information to build models that predict the likely outcome of a patient’s current standing or response to a particular treatment. For chemotherapy, two decisions need to be made: whether or not chemotherapy should be received, and, if so, which one. Many studies have found gene signatures for predicting overall survival or recurrence of breast cancer^4^,^5^,^6^,^7^,^8^,^9^,^10^,^11^,^12^, which can be used to provide guidance on if a more aggressive treatment strategy should be taken. Oncotype DX^®^, a commercially available diagnostic test based on the expression of a 21-gene panel, has been used in the prognosis of breast cancer. Studies have predicted responses for a single treatment or for a patient sample with mixed treatments without stratification by treatment types^13^,^14^,^15^,^16^,^17^,^18^,^19^,^20^,^21^. Cluster analysis has been used to identify subtypes of triple-negative breast cancer patients whose “driver” signaling pathways may be pharmacologically targeted^22^. No studies have developed personalized treatment strategy to select among multiple chemotherapy regimens with an aim of improving the overall response rate. When chemotherapy is to be received, patients still lack guidance on which regimen is the most efficacious for them.

There are two main categories of cytotoxic chemotherapy drugs for breast cancer: anthracyclines and taxanes. Several combinations of the two types of drugs have been used for treating breast cancer, despite the fact that no effective guideline is available for the selection of specific regimen for a patient^2^. For patients who are HER2 negative and ER negative, chemotherapy is still the main therapy of choice. We reanalyzed data collected from seven independent studies with totally 1079 breast cancer patients who received neoadjuvant chemotherapy (Table 1). The data were obtained from GEO database (Gene Expression Omnibus^23^), where clinical information including responses to chemotherapy and gene expression data are available. The responses were coded as pCR (pathologic complete response) or RD (residual disease). pCR is a potential surrogate marker for survival^24^,^25^, a measure for chemosensitivity^9^,^26^, and associated with a favorable outcome^27^,^28^,^29^. pCR has also been used as the primary outcome measure in many clinical trials. Among the 1079 patients, 20.4% of them have pCR and the rest have RD as their responses. Using pCR/RD as the measure of outcome, this study investigated whether the current rate of pCR can be improved by PCT using genomic variables. The patients were divided into three regimen groups according to the treatments given by their oncologists: an anthracycline only (A group), anthracycline plus paclitaxel (TA group), and anthracycline plus docetaxel (TxA group). Our strategy for developing personalized treatment from multiple patient cohorts with different treatments is outlined in Fig. 1.

Random forest models incorporating clinical and genomic variables (clinical-gene-model) were trained for the three groups and assessed in an independent validation setting, in which each of the individual data set was held out in turn as testing data set and the rest of the data sets were used for training. Four genes were found to be significant predictors of pCR for A group, 13 genes for TA group, and 24 genes for TxA group. Based on the predicted responses from the models, we reassigned patients to the regimen that was predicted to have the highest probability of pCR. In the independent validation, the new assignment approach, called PRES (Personalized REgimen Selection), achieved expected rates of pCR, which are significantly higher than those of the best treatments for all seven independent data sets. We further stratified the patients using ER and HER2 status and tested the models using 10-fold cross-validation. PRES is estimated to have pCR rates of 34.4% (95% CI: [31.1%, 39.5%]) for the group who are HER2 negative and 49.2% (95% CI: [44.3%, 56.1%]) for the group who are both HER2 and ER negative. These pCR rates compare favorably to those of the best therapy (TxA, Docetaxel plus Anthracycline), with pCR rates of 30.6% and 41.8% for the two patient groups, respectively. Notably, the improvement for HER2-negative and ER-negative group is quite clinically relevant (7.4% in terms of absolute percentage of improvement and 17.7% relatively). For this patient group, chemotherapy is still the main therapy of choice. In our patient sample, patients who receive TxA regimen generally have higher rate of pCR than those who receive TA regimen and those who receive A regimen. However, to maximize the rate of pCR, regimens should not be selected based on the overall efficacy of the three regimens. Instead, they should be selected based on both patients’ genomic and clinical information.

To validate the discovered genes, we analyzed gene expression data of 18 paclitaxel-sensitive and 3 paclitaxel-resistant triple negative breast cancer cell lines. Our prediction agrees with the drug-sensitivity profile of these cell lines.

In summary, PRES could substantially increase response rates for HER2-negative and ER-negative patients who will receive one of the widely accepted regimens at present for breast cancer treatment.

## Results

### Model performance and gene signatures

The performance of the models for the three types of regimens is tested by 10-fold cross validation (Table S1). Clinical-gene-models generally have better predictive power (higher F_0.5_-scores) than clinical-models. F_0.5_-score is a measure to balance precision and recall with an emphasis on precision^30^. Addition of genomic variables improved the performance for TA and TxA groups dramatically, while the models did not show significant difference for A group. For TA and TxA groups, clinical-gene-models perform much better than clinical-models, indicating genomic variables can be powerful predictors of chemotherapy responses. Based on this comparison, clinical-gene-models are used in the rest of this study. The gene signatures that can effectively predict the treatment responses of each regimen are shown in Table 2.

### Simulation study

To perform the sanity check of our method, we simulated several data sets with known responses using parameters estimated from the real data and tested the performance of our method on the simulated data. The results (Table S2) showed that our method performed well on the simulated data. It has a relatively higher precision and comparable overall performance measured by F_0.5_-score when compared with another commonly used method, LASSO. For optimal regimen selection, precision is more important than recall, as the model with higher precision is always chosen when compared with other models (See Regimen assignment section for more details).

### Performance at different probability intervals and calculation of pCR scores

**Figure 2:**
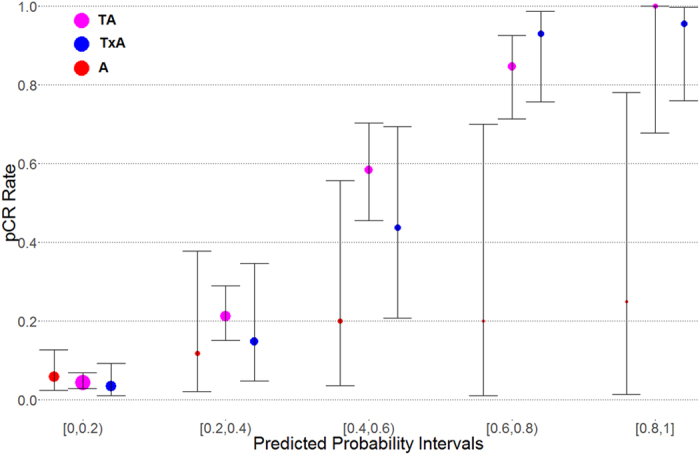
Observed pCR rates vs. predicted probabilities. The predicted probabilities for each regimen are divided into 5 equal length intervals (x-axis). For each interval and each regimen (5 * 3 combinations), the observed pCR rate is calculated by dividing the number of pCR patients with the total number of patients for the particular interval-regimen combination. The predicted probabilities correlate strongly with observed pCR rate in general. However, they differ significantly for some regimens and probability intervals. The three points at each interval for three regimens are scattered around the middle point for visual clarity. The bars show confidence intervals and the sizes of the points are proportional to the number of patients in that particular group.

Since our method predicts the probability of pCR for a treatment given a patient’s clinical and genomic information, it will be interesting to see how the predicted probabilities translate to actual probabilities of pCR. In general, when a predictive model forecasts an outcome with certain probability based on the training data it has used, that probability may not hold true for test data that were not used to train the model. The predicted probabilities of pCR for patients in each regimen group were first sorted and then divided into 5 equally length intervals. In each interval we calculated the observed probability of pCR (number of patients with pCR in that interval divided by the total number of patients in that interval), which are plotted against predicted probabilities of pCR in Fig. 2. The observed probability of pCR in each interval is defined as the pCR score of this interval for the corresponding treatment group. There is a good correlation between predicted probabilities and observed probabilities, but they are not exchangeable generally. The probabilities are skewed since the pCR rate of each regimen is lower than RD rate. We can see that our models perform quite well when the predicted probability of pCR is relatively low or relatively high. The first intervals (predicted probabilities smaller than 0.2) in all the three treatment groups have negative predictive values (NPVs, defined as number of true negatives divided by number of negative calls) of 92% or higher. Models for TA and TxA also perform well on the other end of the spectrum - when the predicted probabilities of pCR are greater than 0.8. In such cases, positive predictive values (PPVs, defined as number of true positives divided by number of positive calls) are also quite high, with 100% for TA and TxA groups. The PPVs are also quite high for probability interval [0.6, 0.8]. For more details on the exact numbers, see Table S4.

### Regimen assignment

Patients are then assigned to the optimal regimen using pCR scores to maximize the expected pCR rate (more details in Methods). Patients, who did not respond to their original regimen, if given to a different regimen according to our models, may have a better chance of pCR. In GSM549310, for example, a patient of age 36, ER-negative, HER2-positive, etc., was originally assigned to A treatment group and the patient did not achieve pCR. If she had received treatment TA, she would have an 84.6% chance of pCR according to our model. The confidence intervals in the last column of Table S5 are calculated from 1000 random samples of pCR scores in Table S4 by approximating the distributions of pCR scores using truncated Gaussian distributions.

### Independent validation to test the performance of PRES

An independent validation study was performed in which each of the seven independent dataset was first left-out when training the model, and then the patients in the left-out dataset was reassigned to their optimal regimens by the model trained using the other six datasets. The performance is shown in Table 3. We can see that our new method, PRES, achieved pCR rates better than the rates of the best treatment from the original assignments for all the seven independent datasets. It strongly indicates that PRES may perform well in real clinical validations.

### Patient stratification based on known biomarkers and clinical information

Breast cancer is a very heterogeneous disease and the differences among the subtypes may not be well characterized by a single model. For HER2-positive patients, trastuzumab (Herceptin) is a quite effective treatment, which is often used in combination with chemotherapy. Similarly, ER-positive patients often receive hormone therapy (endocrine therapy) in combination with chemotherapy. To remove the effect of other confounding therapies, and to understand better the treatment responses of HER2 and ER negative patients, we stratified the patients in our data set using HER2 and ER status. HER2-negative patients (90% of the total patients) and HER2-negative and ER-negative patients (31.4% of the total patients) were studied following the same protocol as the whole dataset. Due to limited sample sizes, HER2-negative and ER-negative patients were studied for patients who received either TA or TxA. The patients who received only anthracyclines (A group) were not included in this study.

**Figure 3:**
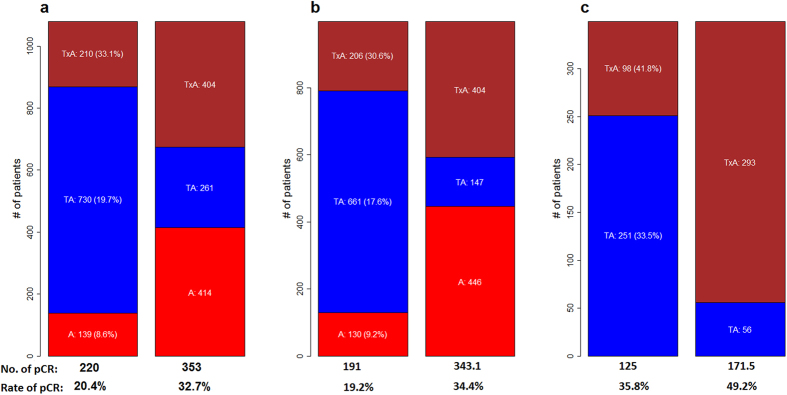
The expected number of pCR and number of patients assigned to each regimen for the whole dataset and different subpopulations. Numbers within the bars are the numbers of patients assigned to the corresponding regimens. Numbers in parenthesis are rate of pCR for the corresponding regimen. In each sub-figure, the bars on the left show numbers from original assignment and those on the right are numbers produced by PRES. (**a**) All patients; (**b**) HER2-negative; (**c**) HER2-negative and ER-negative.

The results for these two studies are also shown in Fig. 3 and Table S5. For the HER2-negative patients, the expected pCR rate is 34.4 (95% CI: [31.1, 39.5]), which is significantly higher than the average pCR rate for the original assignment, 19.2%, and is also higher with statistical significance than the highest pCR rate of the three regimens (TxA group), 30.6%. For HER2-negative and ER-negative patients, the improvement is more dramatic with an expected pCR rate of 49.2 (95% CI: [44.3, 56.1]). The average pCR rate of the original assignment is 35.8% and the highest pCR rate is 41.8%, again from TxA group. The gene signatures obtained for these two studies also share significant number of genes. Detailed results for these two studies are given in Tables S6–S10.

### Validation of paclitaxel (TA) model using cell line data

**Figure 4:**
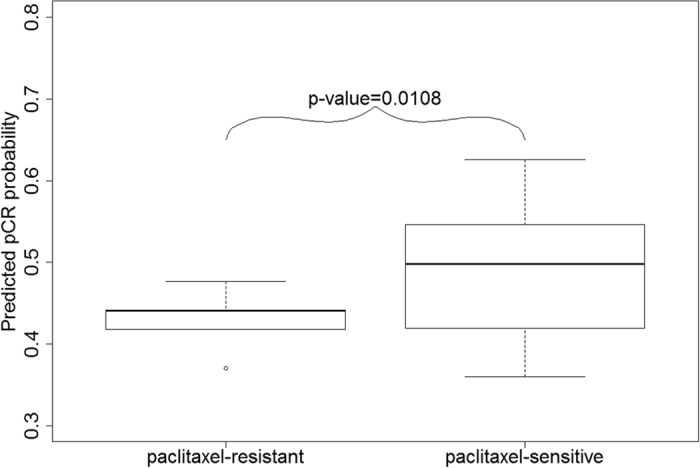
The boxplot for predicted probabilities of paclitaxel-sensitive and resistant groups. The predicted probabilities of pCR for paclitaxel-sensitive cell lines are significantly higher (p-value = 0.0108) than those of the paclitaxel-resistant cell lines.

We used 18 paclitaxel-sensitive and 3 paclitaxel-resistant triple negative breast cancer cell lines^31^ (Table S11) to evaluate the effectiveness of the genes we discovered for predicting the sensitivities of the cell lines to paclitaxel. The hypothesis to be tested is *H*_0_:*P*_*resist*_ = *P*_*sensitive*_ against *H*_1_:*P*_*resist*_ < *P*_*sensitive*_, where *P*_*resist*_ and *P*_*sensitive*_ represent the mean probabilities of the resistant cell lines to achieve pCR and the sensitive cell lines to achieve pCR, respectively. The gene expression data were obtained from two separate studies (GSE10890 and GSE34211)^32^,^33^,^34^,^35^. We computed *P*_*resist*_ and *P*_*sensitive*_ using the gene expression data and performed Welch two sample t-test, which gave a p-value of 0.0108. The predicted probabilities of achieving pCR for the sensitive cell lines are significantly higher than those of the resistant ones (Fig. 4).

### Comparison between paclitaxel (TA) and docetaxel (TxA)

Several clinical trials have shown the benefit of addition of taxanes to anthracycline-based regimens^36^. Both Paclitaxel and docetaxel belong to taxanes family of anti-cancer compounds, and share major parts of their structures and mechanisms of action. However, they differ in several aspects including depolymerization inhibition activity and toxicity profiles^37^. Paclitaxel and docetaxel, when administered as a single agent, have similar efficacy to anthracyclines in patients naive to chemotherapy^38^,^39^. Several clinical trials also showed that the improvements in DFS (disease-free survival) and OS (overall survival) were similar for both paclitaxel and docetaxel when combined with anthracyclines^40^. In our combined dataset, more patients in TxA regimen group have pCR (30.5%) than those in TA group (19.7%). Of course, that does not necessarily serve as a strong evidence for docetaxel having higher efficacy than paclitaxel. A key question that remains to be answered is: do patients react very similarly to both drugs? Are there sub-populations of patients who should receive one drug in preference to the other? As both paclitaxel and docetaxel are commonly used for breast cancer treatment, this is a question with significant clinical implications. In this study, the comparison between paclitaxel and docetaxel was performed using HER2-negative and ER-negative patients. We can see from Fig. 3 that although TxA regimen gives better efficacy than TA regimen in general (19.7% for TA vs 30.5% for TxA), a substantial number of patients (261 patients in Fig. 3a) responds better to TA regimen. To maximize the rate of pCR, one should select regimen according to the characteristics of each patients.

## Discussion

Given the currently available regimens for breast cancer patients, how much can personalized cancer therapy (PCT) using genomic information further improve the response rates we have achieved so far? To address this question, we used high-throughput gene expression data from seven independent studies with totally 1079 breast cancer patients who received neoadjuvant chemotherapy to investigate whether PCT can improve the rate of pCR for breast cancer patients. The patients fell into three treatment regimen groups: those who received an anthracycline alone (A group), those who received both anthracycline and paclitaxel (TA group), and those who received both anthracycline and docetaxel (TxA group). We found that a substantial number of patients responded differently to at least two regimens (Fig. S2), indicating PCT can be very beneficial for patients who will choose one of these regimens. We also found that the variable selection method we designed can select a small number of genes that can effectively differentiate the patients who will have higher probability of pCR under a certain regimen. We designed a PCT strategy, PRES (Personalized REgimen Selection), and applied it retrospectively to the patients in our data set. An independent validation test showed that the pCR rate can be significantly improved for all the seven independent datasets used in this study. When patients were stratified using ER and HER2 status, we found that the pCR rate can be potentially improved from 19.2% to 34.4% (95% CI: [31.1%, 39.5%]) for HER2-negative patients, and from 35.8% to 49.2% (95% CI: [44.3%, 56.1%]) for HER2-negative and ER-negative patients. When compared to the regimen with the highest pCR, the improvement was also significant for HER2-negative patients (30.6% from TxA regimen), and highly significant for HER2-negative and ER-negative patients (41.8% from TxA regimen). Our study found that 11.2% of patients were likely overtreated, meaning they received TA or TxA regimen, but if they had received A regimen they would have had at least the same probability of pCR. The study also found that 5.1% patients were undertreated, meaning that they received A regimen, but if they had received TA or TxA, they would had a better probability of pCR. Overall, the current clinical approaches tend to overtreat patients. As the current study is retrospective in nature, a prospective study should be performed to validate the conclusions drawn from this study.

PRES assigned 340 (31.5%) patients to probability intervals with negative predicted values (NPVs) higher than 90% (Table S4), indicating that 31.5% patients will likely have low chance of pCR and their actual chances are indeed low. On the other hand, 63 (5.8%) patients were assigned to the fourth and fifth intervals in TxA regimen with positive predicted values (PPVs) of 84.6% (52 patients) and 100% (11 patients), respectively. An additional group of 52 (4.8%) patients was assigned to the fourth and fifth intervals in TA regimen with PPVs of 93.1% (29 patients) and 95.7% (23 patients), respectively. Taken together, PRES can provide very useful guidance for more than 40% of patients in their decision making.

Our study focuses on optimizing regimen selection for neoadjuvant chemotherapy. Neoadjuvant therapy offers the opportunity of *in vivo* assessment of tumor response as compared to traditional adjuvant therapy. A recent trial has shown that response-guided approach could provide a clinical advantage for the neoadjuvant over the adjuvant approach in early breast cancer^41^. Recently FDA has considered pathologic response to neoadjuvant therapy as an end point to support accelerated drug approval in high-risk, early-stage breast cancer^26^,^42^. These recent developments in breast cancer chemotherapy suggest a more important role of neoadjuvant chemotherapy in breast cancer treatment.

The simulation results reflected that our method had a higher precision while the overall performances were similar to another commonly used method, LASSO. The higher precision is clinically important since higher precision means lower false positive rate. Precision is also used in optimal regimen selection.

A validation study on 21 breast cancer cell lines showed that our model can effectively differentiate cell lines that are paclitaxel-sensitive from those that are paclitaxel-resistant. This provides a strong support that the set of genes we discovered are able to predict the responses to paclitaxel, and likely to docetaxel, as well.

The approach used in this study can be readily applied to developing personalized cancer therapy for other therapies of breast cancer or for other types of cancers, which will be the subject of our future studies.

## Materials and Methods

### Data

We collected 1079 breast cancer patient samples from 7 data series in GEO database. Samples were divided into 3 treatment groups based on the treatment each patient received: an anthracyline only (A group), anthracycline plus paclitaxel (TA), and anthracycline plus docetaxel (TxA) (Table 1). We used R package Affyio for data normalization. Random forest models were built for each treatment group.

### Model building and evaluation

The overall procedure of PRES is shown in Fig. S1. We first conducted a Welch two-sample t-test to find differentially expressed probes between pCR and RD response groups, using a significance level of 0.05. Then we performed a Random Sampling Screening (RSS) procedure to further narrow down the list of candidate probes.

To determine the optimal number of probes to be included in each model, a 5-fold cross validation is used (more details in supplementary material *Model Building* section). The F_0.5_-score (higher F_0.5_-score means higher predictive ability) was used to measure the performance of the models since the dataset are unbalanced, where many more patients having RD than pCR (details in supplementary material and methods).

### Regimen selection using pCR score to maximize the expected value of pCR rate

Once the models are built for the three regimens, each patient will have a predicted probability of pCR from the model, whose corresponding regimen was the regimen the patient actually received. To avoid over-fitting, the predicted probabilities are obtained using 10-fold cross-validation, meaning that the response of any patient is predicted using the model built without that patient’s information. For each model we sort and divide the predicted probabilities of pCR into 5 probability intervals (PIs) of equal length. We then compute precisions (or positive predicted values) for each interval by taking the ratio of the number of patients with pCR and the total number of patients in the interval. This ratio, called pCR score, is the MLE (maximum likelihood estimator) of the expected value of pCR rate if the predicted probability of pCR for a patient for a particular regimen falls into that particular PI. Note that the predicted probability of pCR by a model is different from the expected value of pCR rate for a patient, which can be considered as the actual probability of pCR a patient should expect to have (Fig. 2). Each regimen has five pCR scores corresponding to the five PIs. Next, we predict the probabilities of being pCR for all the patients under each model. Again, for the patients whose information is used to build a model, their predicted probabilities were obtained from the 10-fold cross-validation. Each patient will have three predicted probabilities of being pCR for the three models built for A group, TA group, and TxA group, respectively. Each probability is then mapped to one of the PIs for each model. The regimen, whose mapped PI has the highest pCR score, will be the optimal regimen assigned to the patient. To take toxicities of the regimens into account, if the pCR score for A treatment is within +/− 0.01 of the pCR score of the other two regimens, we assign the patient to A treatment. We assigned a treatment to each patient (instead of leaving some patients “untreated”) for the following reasons: (1) if we do not assign any treatment to some patients because they respond poorly to all the regimens, the overall response rate will be lower. Since every patient was assigned a treatment in the original data set, the comparison will not be fair; (2) the response rate will be further affected by the pCR score cutoff we use to determine whether a regimen should be assigned to a patient, which can be rather subjective; and (3) in practice, even patients know they will not respond well to any regimens, they may still choose the one from which they will benefit the most.

Alternatively, we also performed assignment with the purpose of achieving the highest pCR score without consideration of toxicity, and obtained slightly higher expected rate of pCR. The expected number of pCR cases is computed as the sum of pCR scores of all the patients based on the regimens assigned to them.

## Additional Information

**How to cite this article**: Yu, K. *et al*. Personalized chemotherapy selection for breast cancer using gene expression profiles. *Sci. Rep.* **7**, 43294; doi: 10.1038/srep43294 (2017).

**Publisher's note:** Springer Nature remains neutral with regard to jurisdictional claims in published maps and institutional affiliations.

## Supplementary Material

Supplementary Materials

## Figures and Tables

**Figure 1:**
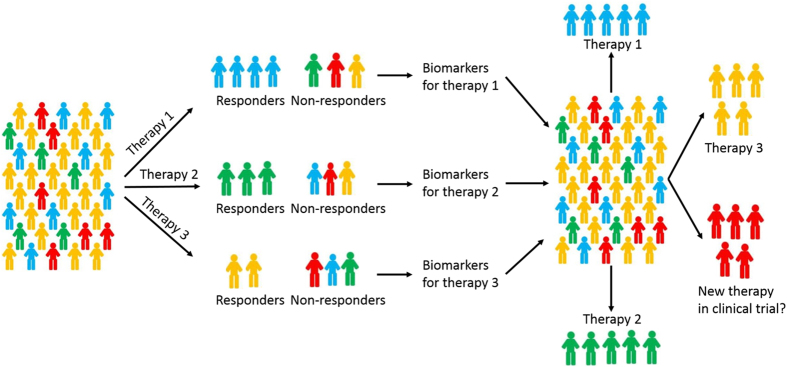
Schematic illustration of our strategy for developing personalized treatment from multiple patient cohorts with different treatments. All the patients received one of three therapies. Each group has responders and non-responders. From each treatment group, we identify biomarkers and build predictive models for selecting responders for the corresponding treatment. The three models are validated through cross-validation, where each patient is evaluated using the model trained without using that patient’s information. To assess the overall performance of the three sets of biomarkers and corresponding models, all the patients are evaluated by all the three models and the therapy with the highest probability of giving pathological complete response (pCR) is assigned to the patient. The expected probability of pCR is calculated and compared with the actual pCR, which can be either the average pCR of the three regimens or the highest pCR of the three regimens. Here all the patients are assigned a therapy for comparison purpose since all the patients in reality received one of the three therapies. In practice, patients who are predicted to not respond well to any of the therapies may opt not taking any of them and try a new therapy. Note, the numbers of colored human figures have no actual meaning. In reality, there are patients who respond to more than one regimen. The responders in this figure represent those who have the best response for the corresponding regimen.

**Table 1:** GEO data sets used in the study and number of patients in each data set.

GEO accession number	Regimen			Total
	Anthracycline (A)	Paclitaxel and Anthracycline (TA)	Docetaxel and Anthracycline (TxA)	
GSE20194^43^	4 (0)	257 (20.6%)	8 (12.5%)	269 (20.1%)
GSE20271^44^	85 (8.2%)	91 (20.9%)	—	176 (14.8%)
GSE22093^45^	50 (10%)	—	—	50 (10%)
GSE23988^45^	—	—	61 (32.8%)	61 (32.8%)
GSE25055^13^	—	290 (18.3%)	—	290 (18.3%)
GSE25065^13^	—	92 (20.7%)	88 (26.1%)	180 (23.3%)
GSE42822^46^	—	—	53 (37.7%)	53 (37.7%)
Total	139 (8.6%)	730 (19.7%)	210 (30.5%)	1079 (20.4%)

Values in parenthesis are percentage of patients who have pCR among the patients in the corresponding regimen group. The rest of the patients have RD. All the patients were put into one of three regimen groups based on the treatment each patient received: anthracycline alone (A), paclitaxel and anthracycline (TA), and docetaxel and anthracycline (TxA).

**Table 2:** Genes selected for the three regimens. Multiple probes are selected for some genes (e.g. NFIB and H2AFZ).

Probe Set	Symbol	Description	Chromosome	pCR Status*
Anthracycline (A) regimen				
218066_at	SLC12A7	solute carrier family 12 (potassium/chloride transporter), member 7	5	−
210164_at	GZMB	Granzyme B (Granzyme 2, Cytotoxic T-Lymphocyte-Associated Serine Esterase 1)	14	+
213211_s_at	TAF6L	TAF6-Like RNA Polymerase II, P300/CBP-Associated Factor (PCAF)-Associated Factor, 65 kDa	11	−
214567_s_at	XCL2	Chemokine (C Motif) Ligand 2	1	+
Paclitaxel and anthracycline (TA) regimen				
213033_s_at	NFIB	Nuclear Factor I/B	9	+
219051_x_at	METRN	Meteorin, Glial Cell Differentiation Regulator	16	−
209289_at	NFIB	Nuclear Factor I/B	9	+
205225_at	ESR1	Estrogen Receptor 1	6	−
220425_x_at	ROPN1B	Rhophilin Associated Tail Protein 1B	3	+
213032_at	NFIB	Nuclear Factor I/B	9	+
204822_at	TTK	TTK Protein Kinase	6	+
221253_s_at	TXNDC5	Thioredoxin Domain Containing 5 (Endoplasmic Reticulum)	6	+
208712_at	CCND1	Cyclin D1	11	−
221872_at	RARRES1	Retinoic Acid Receptor Responder (Tazarotene Induced) 1	3	+
203693_s_at	E2F3	E2F transcription factor 3	6	+
204825_at	MELK	Maternal embryonic leucine zipper kinase	9	+
206754_s_at	CYP2B7P	Cytochrome P450, Family 2, Subfamily B, Polypeptide 7, Pseudogene	19	+
Docetaxel and anthracycline (TxA) regimen				
203554_x_at	PTTG1	pituitary tumor-transforming 1	5	+
202107_s_at	MCM2	minichromosome maintenance complex component 2	3	+
200934_at	DEK	DEK Proto-Oncogene	6	+
200853_at	H2AFZ	H2A histone family, member Z	4	+
210052_s_at	TPX2	TPX2, microtubule-associated, homolog (Xenopus laevis)	20	+
202825_at	SLC25A4	Solute Carrier Family 25 (Mitochondrial Carrier; Adenine Nucleotide Translocator), Member 4	4	−
201930_at	MCM6	minichromosome maintenance complex component 6	2	+
202427_s_at	BRP44	brain protein 44	1	+
218437_s_at	LZTFL1	Leucine Zipper Transcription Factor-Like 1	3	−
212695_at	CRY2	Cryptochrome Circadian Clock 2	11	−
201853_s_at	CDC25B	cell division cycle 25 homolog B (S. pombe)	20	+
201695_s_at	PNP	purine nucleoside phosphorylase	14	+
208079_s_at	AURKA	Serine/Threonine-Protein Kinase Aurora-A	20	+
204159_at	CDKN2C	Cyclin-Dependent Kinase Inhibitor 2 C	1	+
202633_at	TOPBP1	DNA Topoisomerase II-Beta-Binding Protein 1	3	+
207618_s_at	BCS1L	BC1 (Ubiquinol-Cytochrome C Reductase) Synthesis-Like	2	−
212055_at	C18orf10	Tubulin Polyglutamylase Complex Subunit 2	18	+
202951_at	STK38	Serine/Threonine Kinase 38	6	+
201896_s_at	PSRC1	Proline and Serine Rich Coiled-Coil 1	1	+
214435_x_at	RALA	V-Ral Simian Leukemia Viral Oncogene Homolog A (Ras Related)	7	+
208920_at	SRI	Calcium Binding Protein Amplified In Mutlidrug-Resistant Cells	7	−
204767_s_at	FEN1	Flap Structure-Specific Endonuclease 1	11	+
210648_x_at	SNX3	Sorting Nexin 3	6	+
216248_s_at	NR4A2	Nuclear Receptor Subfamily 4 Group A Member 2	2	−
204900_x_at	SAP30	Sin3A Associated Protein 30 kDa	4	+
204822_at	TTK	Phosphotyrosine Picked Threonine-Protein Kinase	6	+
214456_x_at	SAA1/2	Serum Amyloid A1/2	11	+
203418_at	CCNA2	Cyclin A2	4	+
207175_at	ADIPOQ	Adiponectin, C1Q And Collagen Domain Containing	3	+
221599_at	C11orf67	Adipogenesis Associated, Mth938 Domain Containing	11	−

*pCR status: “+”, gene expression up-regulated in pCR cases; “−”, gene expression down-regulated in pCR cases.

**Table 3:** PRES assignment and expected pCR for each study in the independent validation, where each independent data set being tested was left out when training the models.

Study	TA	TxA	A	pCR rate (%)
20194 original	257 (20.6%)	8 (12.5%)	4 (0)	20.1
**20194 PRES**	**139**	**101**	**29**	**29**.**5** (**25**.**7**, **34**.**8**)
20271 original	91 (20.9%)	—	85 (8.2%)	14.8
**20271 PRES**	**36**	**49**	**91**	**29**.**7** (**23**.**5**, **46**.**6**)
22093 original	—	—	50 (10%)	10
**22093 PRES**	—	**50**	—	**15**.**6** (**11**.**4**, **19**.**5**)
23988 original	—	61 (32.8%)	—	32.8
**23988 PRES**	**17**	**44**	**0**	**45**.**6** (**39**.**1**, **53**.**1**)
25055 original	290 (18.3%)	—	—	18.3
**25055 PRES**	**0**	**290**	**0**	**41**.**1** (**33**.**4**, **49**.**4**)
25065 original	92 (20.7%)	88 (26.1%)	—	23.3
**25065 PRES**	**18**	**160**	**2**	**37**.**0** (**29**.**1**, **46**.**7**)
42822 original	—	53 (37.7%)	—	37.7
**42822 PRES**	**15**	**38**	**0**	**45**.**3** (**39**.**1**, **52**.**2**)
