# Supplementary material for: Personalized chemotherapy selection for breast cancer using gene expression profiles
Source: Sci Rep. 2017 Mar 3;7:43294. doi: 10.1038/srep43294 (PMC5335706; doi:10.1038/srep43294)
Supplement: Supplementary Materials [file srep43294-s1.pdf]

Personalized chemotherapy selection for breast cancer using gene expression profiles

Authors: Kaixian Yu[1*], Qing-Xiang Amy Sang[2], Pei-Yau Lung[1], Winston Tan[3], Ty Lively[2], Cedric Sheffield[2], Mayassa J. Bou-Dargham[2], Jun S. Liu[4*], Jinfeng Zhang[1*]

Supplementary materials

Model building and evaluation. The overall procedure of PRES is shown in Fig. S1. We first conducted a Welch two-sample t-test to find differentially expressed probes between pCR and RD response groups. Using a significance level of 0.05, the set of significant probes were selected as S_0. We then performed a Random Sampling Screening (RSS) procedure on S_0 as described below to further narrow down the list of candidate probes:

1. Randomly sampling certain amount (pre-specified, in our study we use a quarter of the size of S_0) of probes from S_0 as C_0;
2. Training a random Forest model using probes in C_0 to select a relatively important (ranked by importance, the importance here is defined as area under curve change(46)) subset of probes as $R_0^{(1)}$ (The size of $R_0^{(1)}$ could be either predefined or determined by cross validation, in our study the latter technique was adopted);
3. Repeating (1) and (2) K times, recording all the probes that appeared in $R_0^{(1)}$,..., $R_0^{(K)}$ as S_1 (We used K=1000 in our study);
4. Replacing S_0 with S_1, redo (1), (2), and (3); in (3), instead of keeping all probes appeared, we now keep only the ones with occurrence rate (the ratio of times being selected and times being sampled) over 50%;
5. Repeating (4) until some iteration *n* where the size of S_n is either the same as S_{n-1} or smaller than a predefined number (50 as default). S_n is the final set of probes discovered by RSS.
6. Training random Forest using probes in S_n then use importance to rank the probes in S_n.

Because our datasets are unbalanced (more patients with RD than pCR), we used $F_{0.5}$-score, positive precision and positive recall to measure model performance. $F_{0.5}$-score is defined as $(1+0.5^2)\times$precision$\times$recall/(0.5^2*precision + recall), where precision is defined as (number of patients who are predicted to be pCR and observed to be pCR)/(number of patients who are predicted to be pCR), and recall is defined as (number of patients who are predicted to be pCR and observed to be pCR)/(number of patients who are observed to be pCR). $F_{0.5}$-scores were calculated from a 5-fold cross-validation, where we conducted RSS on each training fold to obtain the candidate sets: S_{n1}, ..., S_{n5}. To select significant probes to a model and evaluate the model, we first added the probes one at a time (from highest ranked) to the clinical-model with only clinical variables (age, ER-status, HER2-status, t-stage, and n-stage). Then we recorded $F_{0.5}$-score along the path. The optimal number of probes for the model was chosen to be the number of probes corresponding to the maximum $F_{0.5}$-score for first *N* probes (we used N=30).

Simulation study

To generate simulated data, first the pCR to RD ratio was set to be 200:800 and 100:900 (pCR:RD) to mimic the situation that in the real data in which there are more RD than pCR. For the predictors, 10 informative predictors ($X_1, \dots, X_{10}$) 990 non-informative predictors ($X_{11}, \dots, X_{1000}$) were included, and three scenarios were considered:

1). All predictors were independent and uncorrelated, there was a mean upshift for K samples (100 or 200) and downshift for 1000-K samples for the informative predictors, but the means were 0 for all non-informative predictors, that is

$$x_{ij} \sim \begin{cases} N\left(0.5I_{j\le K} - 0.5I_{j>K}, 1\right), & i \le 10\} \\ N(0,1) & , \quad i > 11 \end{cases},$$

Where j represents the jth sample.

2). Like 1) but the informative predictors are correlated,

$$x_j \sim MVN\ (\mu, \Sigma)$$

Where$\mu_{ij} = 0.5I_{j\le K\ and\ i\le 10} - 0.5I_{j>K\ and\ i\le 10}$, and $\Sigma_{mn} = (-0.9)^{|m-n|}$ (Σ_{mn} is the entry on the mth row and nth column of Σ)

3) based on 1), but we imposed an interaction pattern: $(X_1 + X_2) * (X_3 + X_4)$

The responses were naturally assigned as 1 or -1 (pCR or RD respectively). We compared our method with LASSO on logistic regression. 10-fold cross validation was used to evaluate the performances.

Cell line validation

We collected data for 21 cell lines (some with replicates), among which 18 are paclitaxel sensitive and 3 are paclitaxel resistant (Table S17).

We used our model to predict the probabilities of these cell lines to be pCR, then test the hypothesis

$$H_0: P_{resist} = P_{sensitive}$$
$$H_1: P_{resist} < P_{sensitive},$$

where P_{resist} and $P_{sensitive}$ represent the mean probabilities of the resistant cell lines to achieve pCR and the mean probabilities of the sensitive cell lines to achieve pCR, respectively. A Welch's two sample t-test gives the p-value 0.0108; therefore, we reject the null hypothesis. One could also tell the probabilities being pCR of the paclitaxel sensitive group is significantly higher than the ones of the paclitaxel resistant group from the boxplots (**Figure 2**) for the two groups.

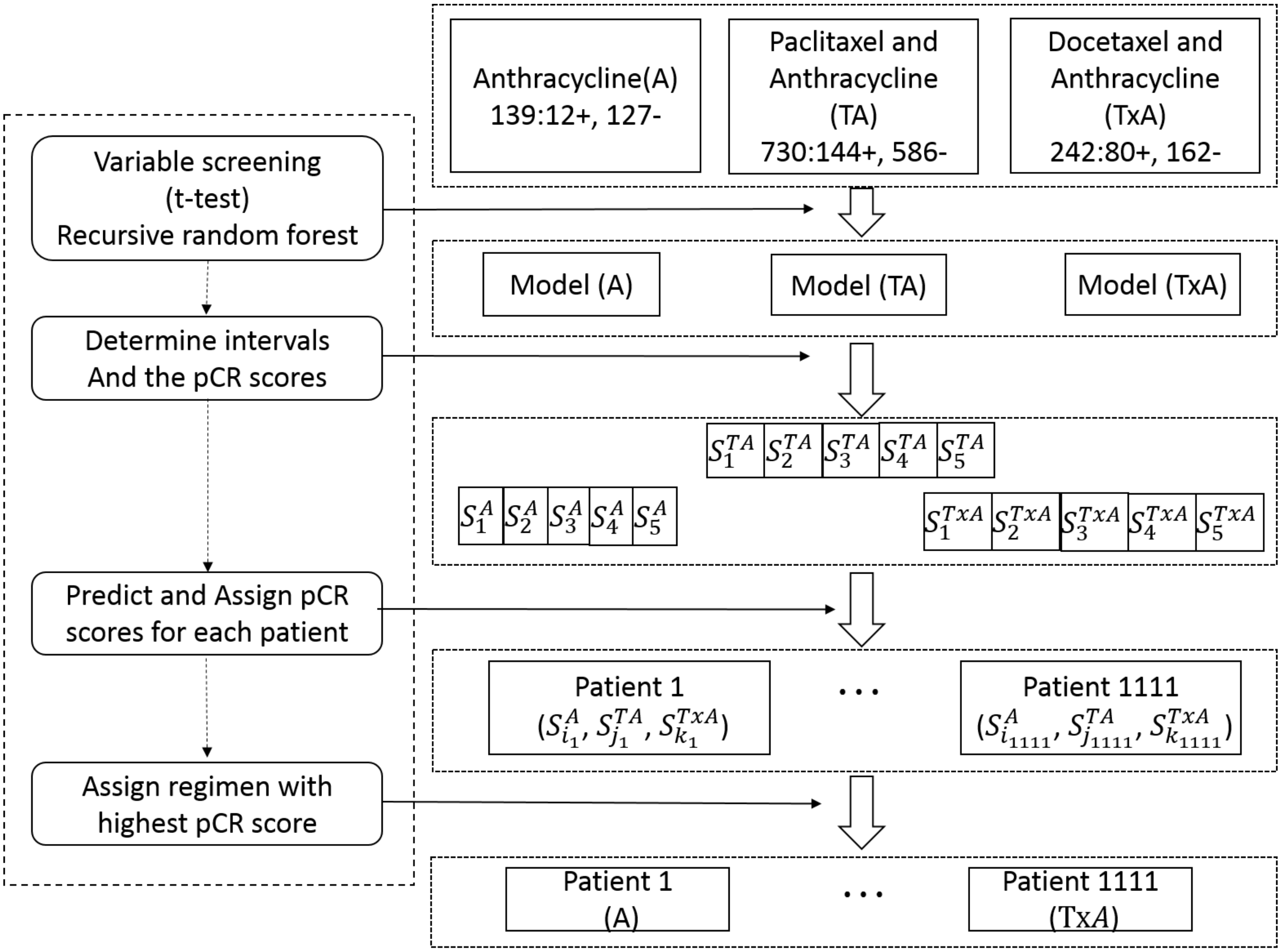

Fig. S1. The procedure of PRES. The numbers in the regimen boxes (i.e. 139:12+, 127-, etc.) are number of patients, patients with pCR, and patients with RD in the corresponding regimen group.

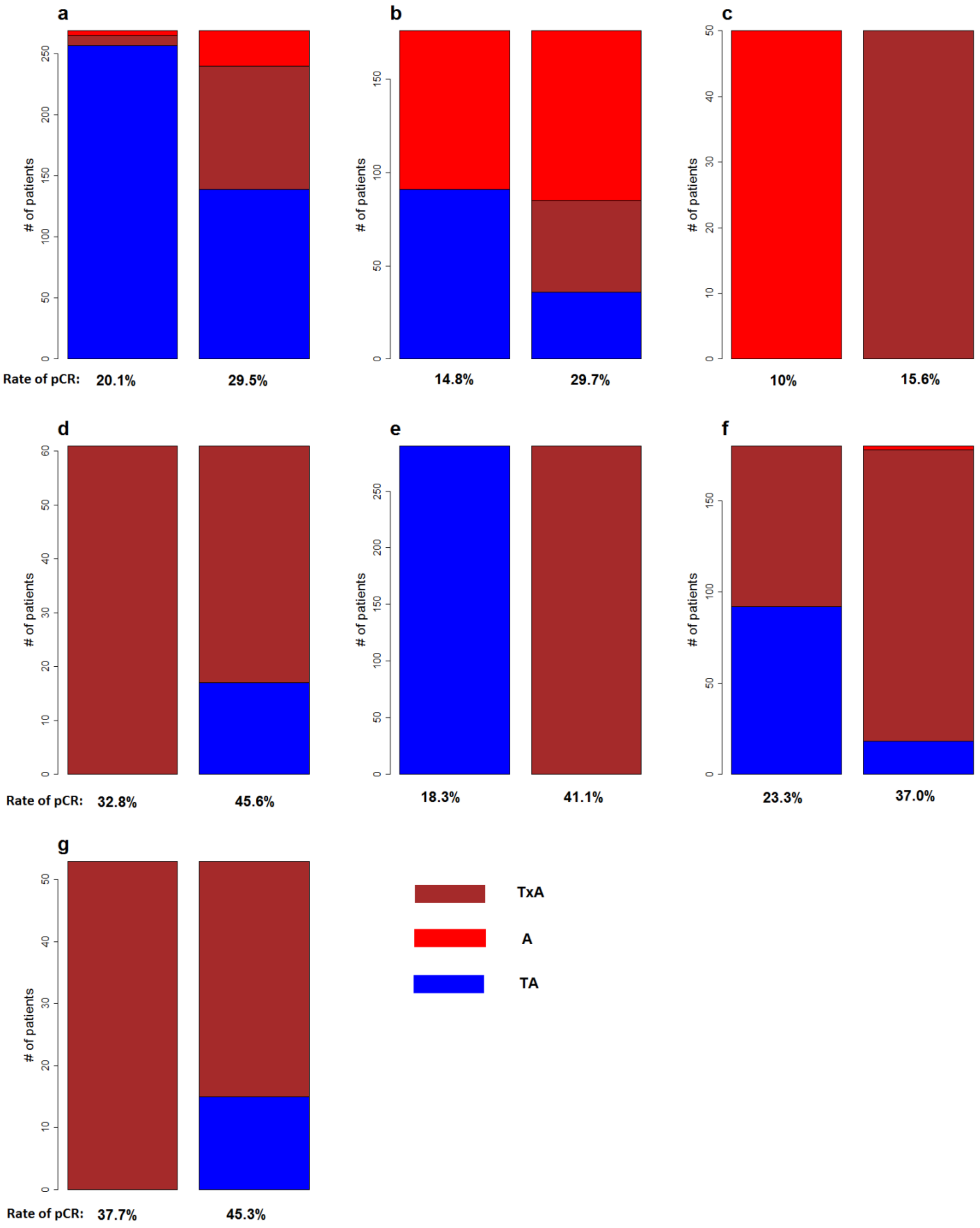

Fig. S2. The expected pCR and assignment for each study. **(a).** GSE20194, **(b).** GSE20271 **(c).** GSE22093, **(d).** GSE23988, **(e).** GSE25055, **(f).** GSE25065, **(g).** GSE42822. The left side of each bar plot is the original assignment, and the right side is the PRES assignment

Table S1. The performance of the models, based on 10-fold cross validation. Clinical-model: only clinical variables are used, clinical-gene-model: both clinical and genomic variables. A: anthracyclines only, TA: anthracycline and paclitaxel, TxA: anthracycline and docetaxel.

Regimens	clinical-model			clinical-gene-model		
	$F_{0.5}$-score	Precision	Recall	$F_{0.5}$-score	Precision	Recall
TA	0.457	0.652	0.208	0.716	0.79	0.522
TxA	0.547	0.565	0.487	0.891	0.938	0.860
A	0.367	0.4	0.35	0.320	0.333	0.278

Table S2. The simulation result for model building.

	Recursive Random Forest			LASSO		
	pCR:RD = 200:800					
	Precision	Recall	$F_{0.5}$-score	Precision	Recall	$F_{0.5}$-score
No correlation	0.942	0.734	0.891	0.927	0.824	0.904
Correlation	0.932	0.814	0.906	0.924	0.857	0.910
Interaction	0.857	0.744	0.832	0.838	0.763	0.822
	pCR:RD = 100:900					
	Precision	Recall	$F_{0.5}$-score	Precision	Recall	$F_{0.5}$-score
No correlation	0.938	0.512	0.804	0.924	0.696	0.867
Correlation	0.933	0.813	0.906	0.924	0.857	0.910
Interaction	0.855	0.742	0.830	0.837	0.761	0.821

Table S3. Independent validation. For each regimen of one of the seven independent studies, we used all the data except the data from this study to train the model, we then test the model on this left-out study.

Study	Patients(pCR rate)	$F_{0.5}$-score	Precision	Reall
Anthracycline (A)				
20194	4 (0)	na	na	na
20271	85 (8.2%)	0.450	1.000	0.143
22093	50 (10%)	0.200	0.200	0.200
Paclitaxel and Anthracycline (TA)				
20194	257 (20.6%)	0.791	0.903	0.528
20271	91 (20.9%)	0.678	0.800	0.421
25055	290 (18.3%)	0.681	0.725	0.547
25065	92 (20.7%)	0.484	1.000	0.158
Docetaxel and Anthracycline (TxA)				
20194	8 (12.5%)	na	Na	Na
23988	61 (32.8%)	0.976	0.952	1.000
25065	88 (26.1%)	0.759	0.629	0.957
42822	53 (37.7%)	0.950	0.950	0.950

Table S4. Probability intervals and pCR scores for the three regimen groups.

Anthracycline (A)					
Intervals	[0,0.2)	[0.2,0.4)	[0.4,0.6)	[0.6, 0.8)	[0.8,1]
# of patients	103	17	10	5	4
pCR score	0.058	0.118	0.200	0.200	0.250
95%CI	(0.024,0.128)	(0.021,0.377)	(0.036,0.557)	(0.010,0.702)	(0.013,0.781)
# of patients assigned	340	15	32	20	7
Paclitaxel and Anthracycline (TA)					
Intervals	[0,0.2)	[0.2,0.4)	[0.4,0.6)	[0.6,0.8)	[0.8,1]
# of patients	456	146	65	52	11
pCR score	0.044	0.212	0.585	0.846	1
95%CI	(0.027,0.069)	(0.150,0.290)	(0.455,0.704)	(0.713,0.927)	(0.678,1]*
# of patients assigned	0	143	138	98	25
Docetaxel and Anthracycline (TxA)					
Intervals	[0,0.2)	[0.2,0.4)	[0.4,0.6)	[0.6,0.8)	[0.8,1]
# of patients	115	27	16	29	23
pCR score	0.035	0.148	0.438	0.931	0.957
95%CI	(0.011,0.092)	(0.048,0.347)	(0.207,0.695)	(0.757,0.988)	(0.760,0.998]
# of patients assigned	0	126	76	48	11

* The 95% confidence interval for TA regimen interval [0.8, 1] and TxA regimen interval [0.8, 1] are assigned conservatively using Rule of three *(45,46)*.

Table S5. The expected number of pCR and number of patients assigned to each regimen for the whole dataset and different stratifications. HER2-: HER2-negative, ER-: ER-negative. A: anthracycline alone regimen, TA: paclitaxel and anthracycline regimen, TxA: docetaxel and anthracycline regimen.

Strata	Regimens								
	Assignment	Model performance for A[a]	# patients assigned to A[b]	Model performance for TA	# patients assigned to TA	Model performance for TxA	# patients assigned to TxA	# of pCR[c]	Rate of pCR (%)[e]
All patients (1079)	Original	-	139 (8.6%)	-	730 (19.7%)	-	242 (33.1%)	220	20.4
	**PRES[d]**	**0.320 (0.333)**	**414**	**0.716 (0.79)**	**261**	**0.891 (0.934)**	**404**	**353**	**32.7 (29.1, 37.9)**
HER2- (997)	Original	-	130 (9.23%)	-	661 (17.6%)	-	206 (30.6%)	191	19.2
	**PRES**	**0 (0)**	**446**	**0.758 (0.817)**	**147**	**0.866 (0.887)**	**404**	**343**	**34.4 (31.1, 39.5)**
HER2- & ER- (349)	Original	-	-	-	251 (33.5%)	-	98 (41.8%)	125	35.8
	**PRES**			**0.729 (0.749)**	**56**	**0.802 (0.794)**	**293**	**172**	**49.2 (44.3, 56.1)**

[a]: $F_{0.5}$-scores (precision or positive predicted value for patients with predicted probability > 0.5) for clinical-gene-models.

[b]: number of patients originally assigned to the regimen or assigned using PRES. Numbers in parenthesis are rate of pCR.

[c]: Number of pCR cases observed based on the original assignment or estimated using PRES (rounded to integers).

[d]: Both pCR score and toxicity, if applicable, are used in regimen selection.

[e]: The rate of pCR. Numbers for Original are the average pCR rates for all regimens. Numbers for PRES are the expected pCR rates. Numbers in parenthesis for PRES are 95% confidence intervals.

Table S6. Model performance for the HER2-negative subpopulation

Group	Clinical variables			Gene and clinical variables		
	$F_{0.5}$-score	Precision	recall	$F_{0.5}$-score	Precision	recall
Anthracycline (A)	0	0	0	0	0	0
Paclitaxel and Anthracycline (TA)	0.326	0.45	0.155	0.758	0.817	0.589
Docetaxel and Anthracycline (TxA)	0.495	0.509	0.444	0.866	0.887	0.790

Table S7. Intervals and pCR scores for HER2-negative subpopulation

Anthracycline (A)					
Interval	[0,0.2)	[0.2,0.4)	[0.4,0.6)	[0.6,1]	[0.8,1]
Counts	101	20	4	1	4
pCR score	0.059	0.200	0.500	0	0
95% CI	(0.024, 0.130)	(0.066, 0.443)	(0.150, 0.850)	(0, 0.945)	(0, 0.604)
Paclitaxel and Anthracycline (TA)					
Interval	[0,0.2)	[0.2,0.4)	[0.4,0.6)	[0.6,0.8)	[0.8,1]
Counts	462	80	63	44	12
pCR score	0.050	0.213	0.460	0.818	0.917
95% CI	(0.032, 0.075)	(0.132, 0.322)	(0.335, 0.590)	(0.667, 0.913)	(0.597, 0.996)
Docetaxel and Anthracycline (TxA)					
Interval	[0,0.2)	[0.2,0.4)	[0.4,0.6)	[0.6,0.8)	[0.8,1]
Counts	110	34	18	19	25
pCR score	0.045	0.147	0.611	0.895	1
95% CI	(0.016, 0.109)	(0.055, 0.319)	(0.361, 0.818)	(0.654, 0.982)	(0.834, 1)

Table S8. Number of patients assigned to each treatment for HER2-negative subpopulation.

Treatment	Paclitaxel and Anthracycline (TA)	Docetaxel and Anthracycline (TxA)	Anthracycline (A)	# of pCR
Original	661	206	130	191*
PRES	147	404	446	343.09
*The original group is observed.				

Table S9. Model performance for HER2-negative and ER-negative subpopulation

Group(# of probes)	Clinical variables			Gene and clinical variables		
	$F_{0.5}$-score	Precision	recall	$F_{0.5}$-score	Precision	recall
Paclitaxel and Anthracycline (TA) (14)	0.357	0.429	0.214	0.729	0.749	0.660
Docetaxel and Anthracycline (TxA) (2)	0.579	0.562	0.659	0.802	0.794	0.833

Table S10. Intervals and pCR scores for HER2-negative and ER-negative subpopulation.

Paclitaxel and Anthracycline (TA)					
Interval	[0,0.2)	[0.2,0.4)	[0.4,0.6)	[0.6,0.8)	[0.8,1]
Counts	102	62	34	38	15
pCR score	0.127	0.145	0.500	0.816	0.933
95% CI	(0.072, 0.212)	(0.073, 0.263)	(0.340, 0.660)	(0.651, 0.917)	(0.660, 0.997)
Docetaxel and Anthracycline (TxA)					
Interval	[0,0.2)	[0.2,0.4)	[0.4,0.6)	[0.6,0.8)	[0.8,1]
Counts	41	16	8	13	20
pCR score	0.195	0.063	0.375	0.692	1
95% CI	(0.093, 0.354)	(0.003, 0.323)	(0.102, 0.742)	(0.388, 0.897)	(0.799, 1]

Table S11. List of all cell lines used in the validation study and their GEO accession.

Paclitaxel sensitive			
Cell line	GEO accession	Cell line	GEO accession
**BT-549**	GSM320598(*47,48*)	**CAL-851**	GSM320617(*47,48*)
**HCC-1937**	GSM320621(*47,48*)	**MDA-MB-157**	GSM1589152(*47,48*)
**MDA-MB-468**	GSM320610(*47,48*)	**SUM159PT**	GSM844706(*49,50*)
**BT-20**	GSM320590(*47,48*)	**HCC-1143**	GSM320631(*47,48*)
**HCC-70**	GSM320625(*47,48*)	**MDA-MB-231**	GSM320604(*47,48*)
**MFM-223**	GSM320634(*47,48*)	**HCC-1806**	GSM320594(*47,48*)
**CAL-148**	GSM320637(*47,48*)	**HCC-1395**	GSM320630(*47,48*)
**HS578T**	GSM320601(*47,48*)	**MDA-MB-436**	GSM320608(*47,48*)
**SUM149PT**	GSM844705(*49,50*)	**MDA-MB-453**	GSM320609(*47,48*)
Paclitaxel resistant			
**CAL-120**	GSM274647(*48*), GSM274665(*48*),GSM275987(*48*)		
**HDQP1**	GSM276024(*48*)	**SW-527**	GSM320640(*47,48*), GSM276036(*48*)

Pathway analysis using Pathway interaction database (PID)

Table S12. A treatment using PID curated data

Pathway Name	Biomolecules in Group	P-value
Caspase Cascade in Apoptosis	GZMB	1.15e-02
IL12-mediated signaling events	GZMB	1.35e-02
Downstream signaling in naïve CD8+ T cells	GZMB	1.43e-02

Table S13. A treatment using Reactome data

Pathway Name	Biomolecules in Group 1	P-value
Activation, myristolyation of BID and translocation to mitochondria	GZMB	8.05e-04

Table S14. TA treatment using PID curated data.

Pathway Name	Biomolecules in Group	P-value
Signaling mediated by p38-gamma and p38-delta	CCND1	4.42e-03
Validated transcriptional targets of AP1 family members Fra1 and Fra2	CCND1	1.48e-02
Trk receptor signaling mediated by PI3K and PLC-gamma	CCND1	1.48e-02
E-cadherin signaling in the nascent adherens junction	CCND1	1.60e-02
FOXM1 transcription factor network	CCND1	1.68e-02

Pathway Name	Biomolecules in Group	P-value
FOXA1 transcription factor network	NFIB	1.75e-02
Integrin-linked kinase signaling	CCND1	1.83e-02
Presenilin action in Notch and Wnt signaling	CCND1	1.83e-02
Notch signaling pathway	CCND1	2.31e-02
ATF-2 transcription factor network	CCND1	2.35e-02
Neurotrophic factor-mediated Trk receptor signaling	CCND1	2.46e-02
Signaling events mediated by focal adhesion kinase	CCND1	2.50e-02
Coregulation of Androgen receptor activity	CCND1	2.50e-02
Regulation of retinoblastoma protein	CCND1	2.66e-02
Validated nuclear estrogen receptor alpha network	CCND1	2.70e-02
Regulation of Telomerase	CCND1	2.74e-02
AP-1 transcription factor network	CCND1	2.82e-02
Validated targets of C-MYC transcriptional repression	CCND1	2.89e-02
Regulation of nuclear beta catenin signaling and target gene transcription	CCND1	3.21e-02
C-MYB transcription factor network	CCND1	3.40e-02

Table S15. TA treatment using Reactome data

Pathway Name	Biomolecules in Group 1	P-value
Cyclin D associated events in G1	CCND1	3.61e-03
RNA Polymerase III Transcription Termination	NFIB	7.22e-03
RNA Polymerase III Transcription	NFIB	7.62e-03
RNA Polymerase III Abortive And Retractive Initiation	NFIB	9.21e-03
Ubiquitin-dependent degradation of Cyclin D1	CCND1	1.95e-02

Table S16. TxA treatment using PID data

Pathway Name	Biomolecules in Group	P-value
ATR signaling pathway	MCM2, MCM7	3.73e-04
Fanconi anemia pathway	USP1	3.83e-02
C-MYB transcription factor network	H2AFZ	6.57e-02

Table S17. TxA treatment using Reactome data

Pathway Name	Biomolecules in Group	P-value
Assembly of the pre-replicative complex	CCNL1, CDT1, MCM2, MCM6, MCM7	3.61e-12

Pathway Name	Biomolecules in Group	P-value
Activation of the pre-replicative complex	CCNL1, CDT1, MCM2, MCM6, MCM7	5.42e-11
DNA Replication Pre-Initiation	CCNL1, MCM2, MCM6, MCM7	1.73e-10
G1/S Transition	CCNL1, MCM2, MCM6, MCM7	4.52e-10
Unwinding of DNA	CCNL1, MCM2, MCM6, MCM7	6.78e-10
Removal of licensing factors from origins	CCNL1, CDT1, MCM2, MCM6, MCM7	3.41e-09
Switching of origins to a post-replicative state	CCNL1, MCM2, MCM6, MCM7	4.22e-07
Regulation of the Fanconi anemia pathway	USP1	1.25e-02
Association of licensing factors with the pre-replicative complex	CDT1	1.25e-02
APC/C:Cdc20 mediated degradation of Securin	PTTG1	6.26e-02
CDT1 association with the CDC6:ORC:origin complex	CDT1	6.38e-02
Orc1 removal from chromatin	CDT1	7.13e-02
